# Supplementary material for: Profiling DNA damage response following mitotic perturbations
Source: Nat Commun. 2016 Dec 15;7:13887. doi: 10.1038/ncomms13887 (PMC5172227; doi:10.1038/ncomms13887)
Supplement: Supplementary Information — Supplementary Figures. [file ncomms13887-s1.pdf]

Mitocheck library
1249 candidate cell division genes
originally identified in HeLa

siRNA Screen 1

- *U-2-OS-H4-mEOS2*
- *Endpoint assay 72h (live cells)*

Selection criteria

- *Nuclear morphology, shape, size*
- *Mitotic figures*
- *Cell death*

129 candidate genes
in U-2-OS with potential mitotic phenotypes
with two independent siRNAs

siRNA Screen 2

- *U2OS-H2B-mCherry*
- *Time-lapse recording 72h*

Selection criteria

- *Mitotic arrest/delay*
- *Mitotic cell death*
- *Aberrant nuclear shape after mitosis*

47 genes
in U-2O-S with validated mitotic phenotypes
with two independent siRNAs

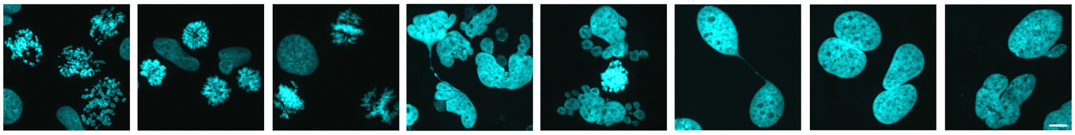

Selected genes cover regulators for major mitotic processes and include:

- *Mitotic kinases*
- *Regulators of mitotic spindle*
- *Regulators of centrosomes*
- *Regulators of sister chromatid cohesion*
- *Regulators of APC/C proteolysis*
- *Regulators of cytokinesis*
- *Regulators of RNA processing*

Supplementary Figure 1. Schematic depiction of siRNA screen for mitotic regulators in U-2-OS cells. Images are DAPI-stained nuclei and illustrate the major mitotic phenotypes obtained after siRNA silencing of the 47 selected genes. Scale bar, 10 µm.

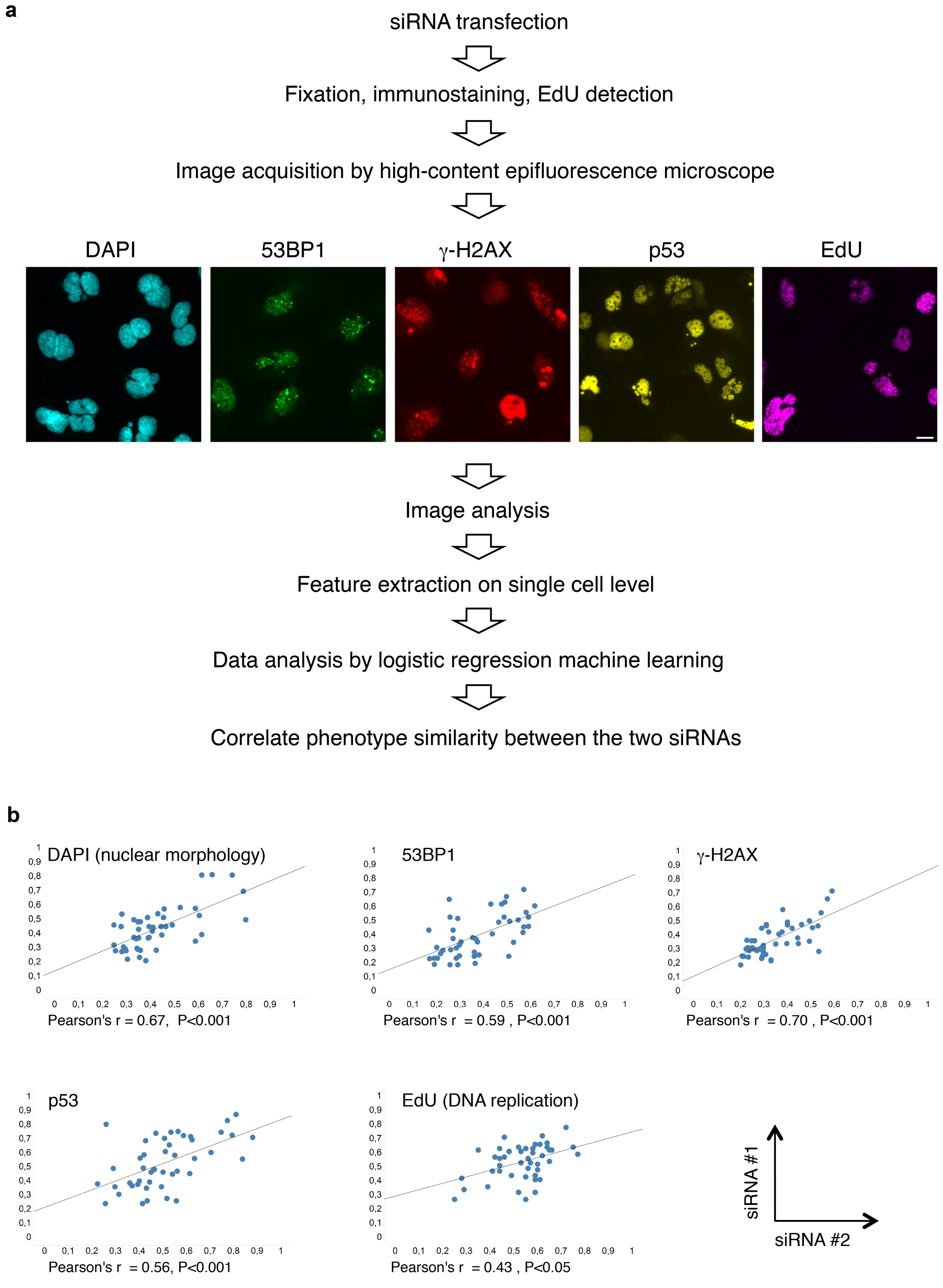

Supplementary Figure 2. (**a**) Schematic depiction of phenotypic profiling of cellular responses to siRNA-mediated silencing of the selected mitotic regulators. Images show examples of the indicated phenotypic markers.

(**b**) Plots depicting comparisons of probabilistic values obtained for each marker with two independent siRNAs. Linear dependence was evaluated by Pearson product-moment correlation coefficient (r) with confidence level (alpha=0.05) and p-values (p) for testing the hypothesis of no correlation. Scale bar, 20 μm.

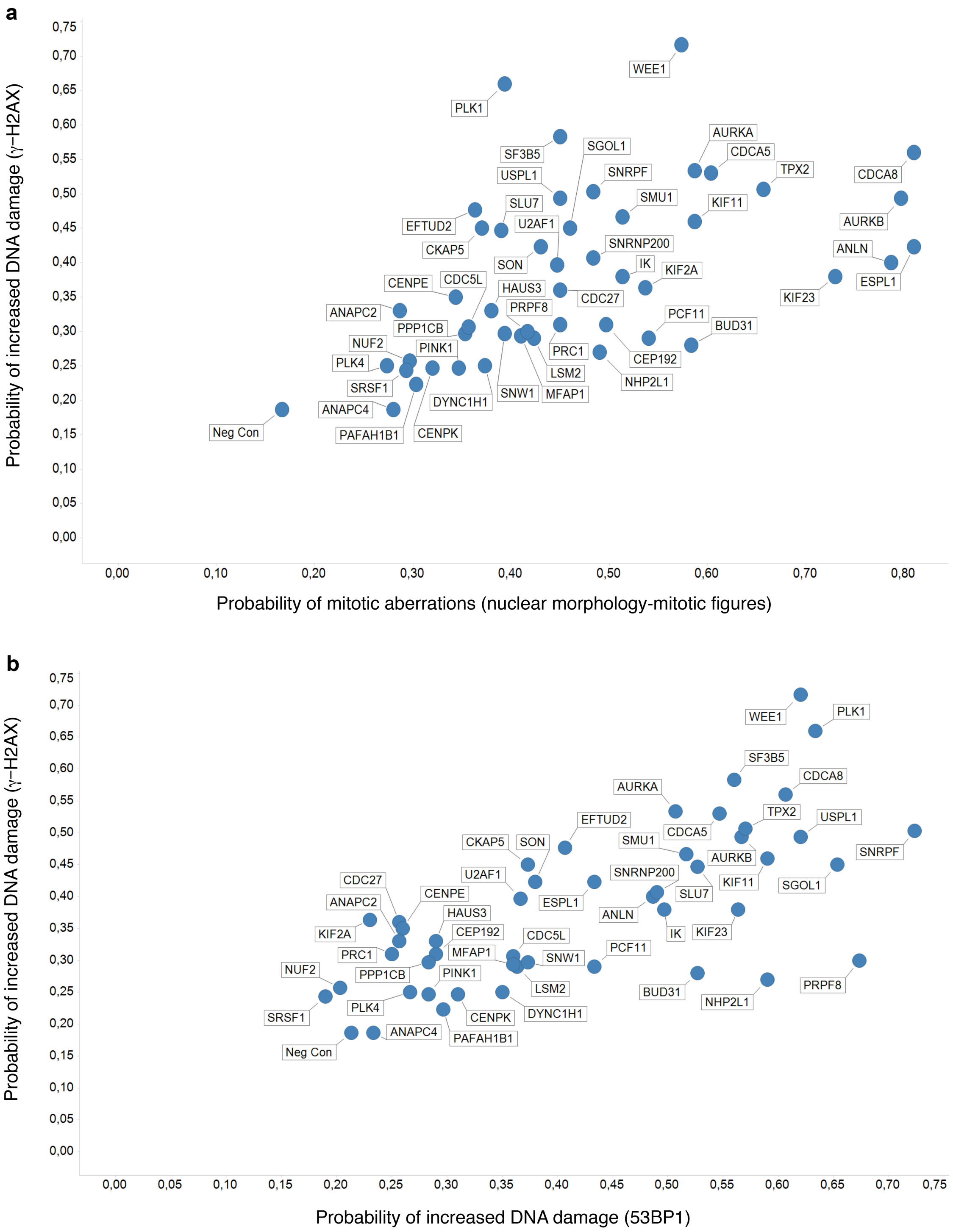

Supplementary Figure 3. DNA damage scored by γ-H2AX recapitulates results with 53BP1. (**a**) Probability scores were generated by logistic regression and are displayed to show correlations between mitotic phenotypes and DDR by γ-H2AX. Values reflect scores derived from n=3 independent siRNA transfections. Data for one of the two siRNAs (siRNA #1) for each depicted target are displayed. Numerical values for both siRNAs and all readouts are listed in Supplementary Dataset 2. (**b**) Direct comparison of probability scores for DNA damage obtained by 53BP1 and γ-H2AX, respectively.

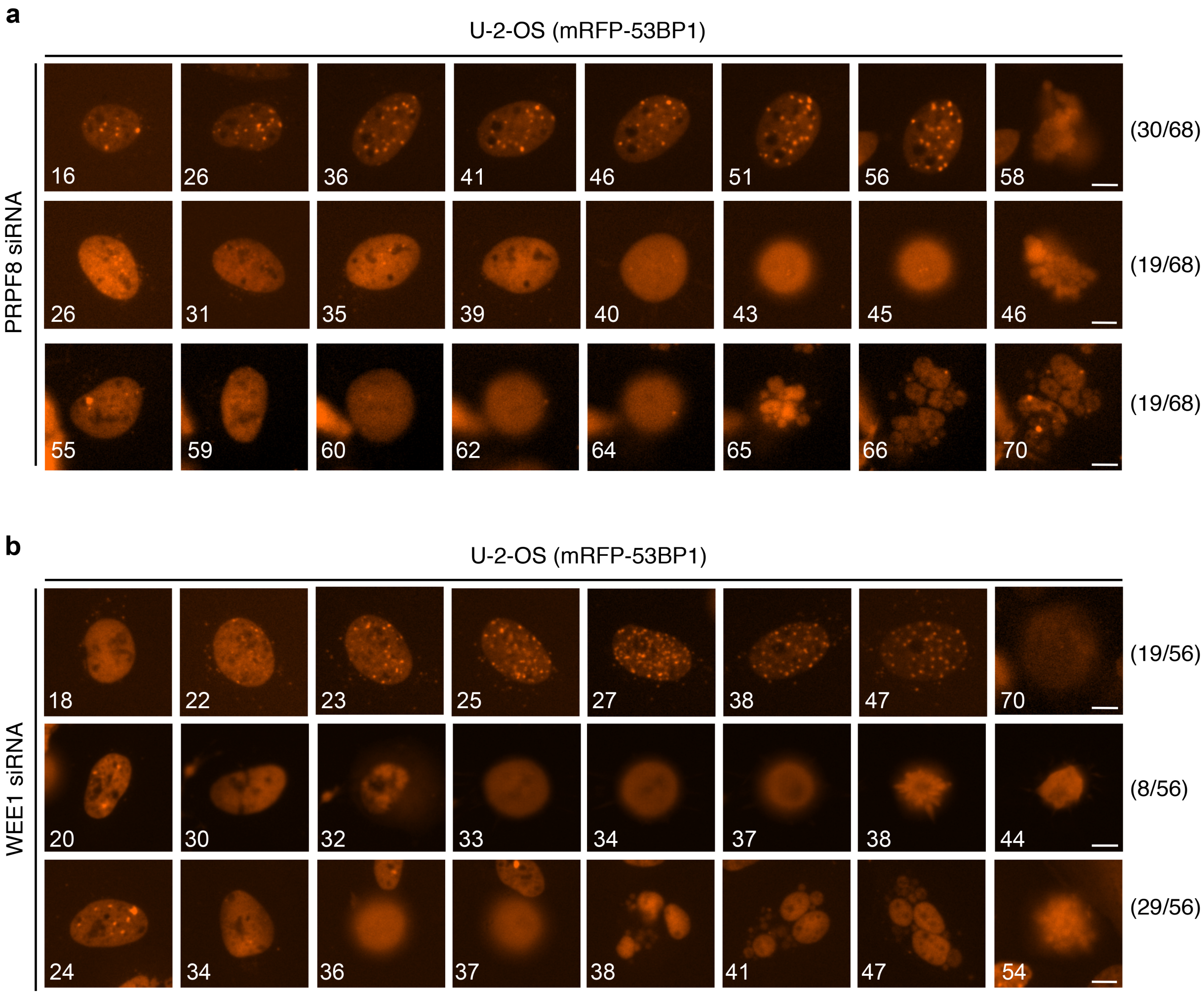

Supplementary Figure 4. Disruption of mRNA splicing and CDK hyperactivation triggers DDR and mitotic errors independent of each other. (**a**) Representative image galleries (n=68) of U-2-OS (mRFP-53BP1) cells treated with siRNA to PRPF8 and subjected to time-lapse microscopy. Numbers indicate time (hours) after siRNA transfection. The frequency of the respective phenotypes is indicated (right). Scale bars, 10 μm. (**b**) Representative image galleries (n=56) of U-2-OS (mRFP-53BP1) cells treated with siRNA to WEE1 and subjected to time-lapse microscopy. Numbers indicate time (hours) after siRNA transfection. The frequency of the respective phenotypes is indicated (right). Scale bars, 10 μm.

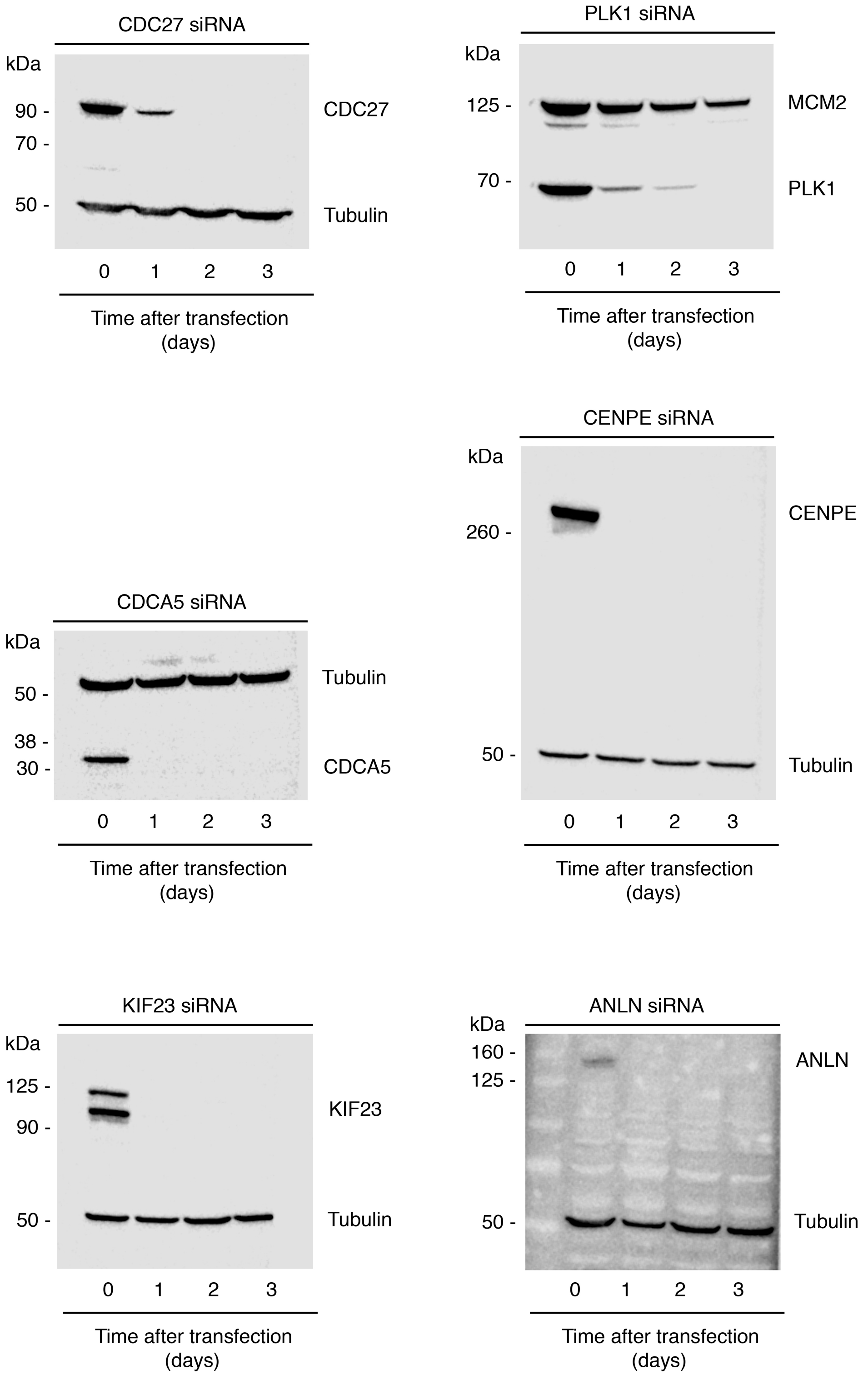

Supplementary Figure 5. Specificity and efficiency of siRNA knockdowns. Western blots of total cell lysates treated with indicated siRNAs and harvested at indicated times after siRNA transfections. Tubulin and MCM2 are shown as loading controls. Uncropped western blots are provided in Supplementary Fig. 6.

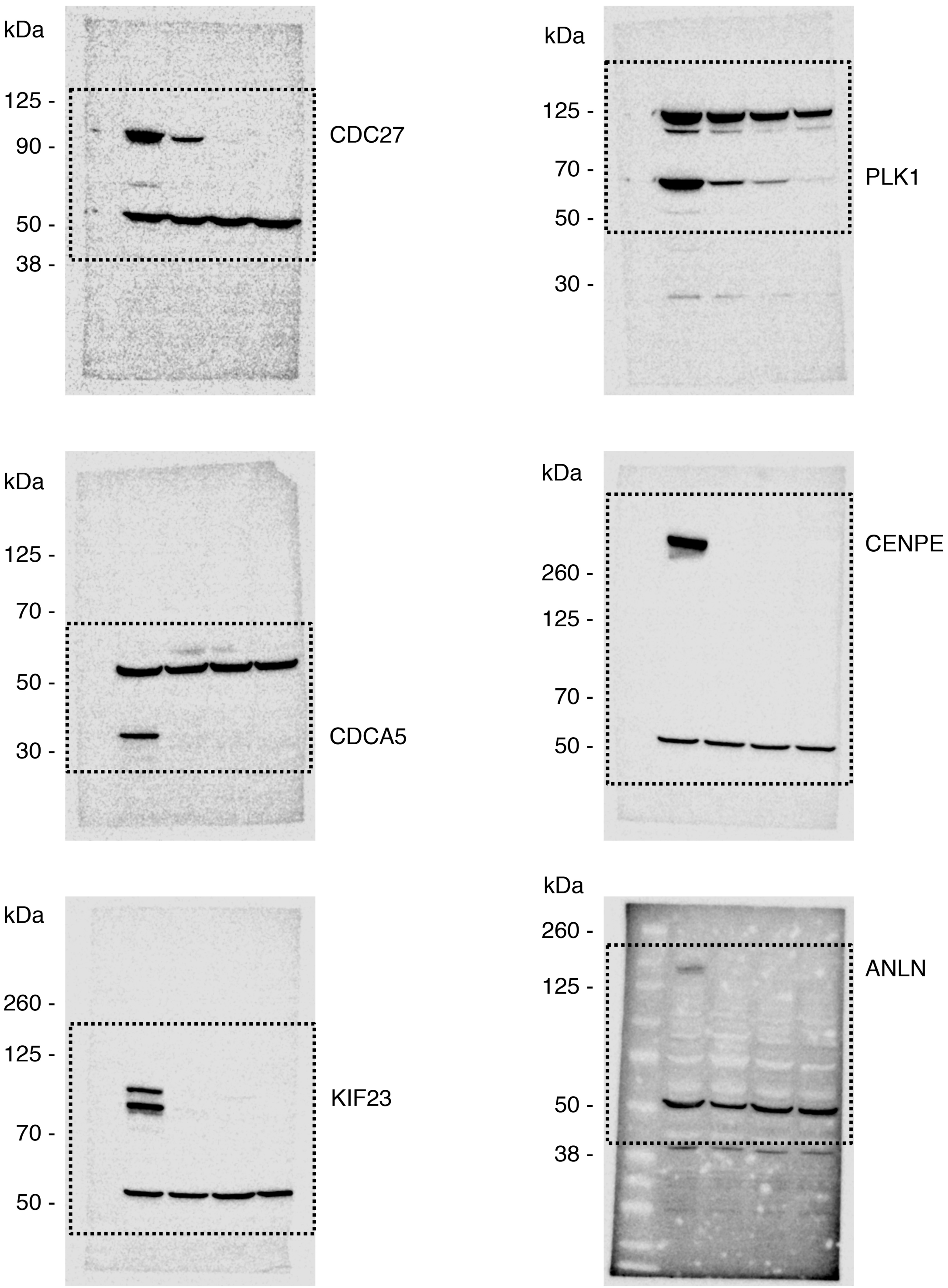

Supplementary Figure 6. Uncropped Western blots. All uncropped images relate to Western blots shown in Supplementary Fig. 5.
